# The XP-Endo Finisher for the removal of calcium hydroxide paste from root canals and from the apical third

**DOI:** 10.4317/jced.53962

**Published:** 2017-07-01

**Authors:** Rami Hamdan, Jérôme Michetti, Déborah Pinchon, Franck Diemer, Marie Georgelin-Gurgel

**Affiliations:** 1 DDS. Service d’Odontologie Conservatrice-Endodontie, Faculté de Chirurgie Dentaire, Toulouse and the CHU de Toulouse, 3 Chemin des Maraîchers, 31400 Toulouse, France; 2 MDS. Service d’Odontologie Conservatrice-Endodontie, Faculté de Chirurgie Dentaire, Toulouse and the CHU de Toulouse, 3 Chemin des Maraîchers, 31400 Toulouse, France and IRIT – UMR CNRS 5505, Avenue de l’étudiant, 31400 Toulouse, France; 3 PHD. Service d’Odontologie Conservatrice-Endodontie, Faculté de Chirurgie Dentaire, Toulouse and the CHU de Toulouse, 3 Chemin des Maraîchers, 31400 Toulouse, France and Institut Clément Ader (ICA, FRE CNRS 3687), 3 Rue Caroline Aigle, 31400 Toulouse, France; 4 PHD. Service d’Odontologie Conservatrice-Endodontie, Faculté de Chirurgie Dentaire, Toulouse and the CHU de Toulouse, 3 Chemin des Maraîchers, 31400 Toulouse, France

## Abstract

**Background:**

The aim was to compare the efficacy of the passive ultrasonic irrigation PUI and the Xp-endo Finisher (FKG-Dentaire, La-Chaux-de-Fonds, Switzerland) in removing the calcium hydroxide paste from root canals and from the apical third.

**Material and Methods:**

Sixty-eight root canals of single-rooted teeth were shaped using the BT-Race files (FKG-Dentaire, La-Chaux-de-Fonds, Switzerland). Ca(OH)2 was placed in all samples except for the negative control group (n=4). Remaining teeth were randomly divided into three groups: G1-Xp (n=30), G2-PUI (n=30) and the positive control group (n=4). Removal procedure consisted of three repeated one-minute-cycles. Samples were split longitudinally, photos of halves were taken at X6.4 magnification and were analyzed using the ImageJ-Software (The National Institutes of Health NIH, Bethesda, Maryland, USA) to calculate the percentage of surfaces with residual Ca(OH)2; the results were compared using the Wilcoxon-Mann Whitney test. Photos of the apical thirds were taken at X16 and X40 magnifications and were scored by two examiners from (0) to (4). Scores of the apical third were compared using the Fisher test.

**Results:**

The Xp-endo Finisher removed completely the Ca(OH)2 dressing from four teeth (13.33%) whereas the PUI in one tooth (3.33%). The mean values of the remaining Ca(OH)2 were (2.1%, 3.6%) respectively and the difference was not significant (*p*= 0.195). Both examiners found the Xp-endo Finisher more efficient in the apical third and the difference was significant; *p*= (0.025, 0.047) respectively.

**Conclusions:**

The Xp-endo Finisher showed a superiority over the PUI in removing the Ca(OH)2 from the apical third after 3 minutes of activation.

** Key words:**Calcium hydroxide removal, Passive ultrasonic irrigation, Xp-endo Finisher.

## Introduction

The complete elimination or reduction of microorganisms is one of the major goals of root canal treatment (1). Root canal instrumentation associated with irrigation is unable to achieve complete cleaning (2). The role of intra-canal medicaments is to enhance the disinfection procedure. Calcium hydroxide Ca(OH)2 is widely used to reduce residual bacteria (3) in spite of its incapability to eradicate all endodontic microorganisms (4). It is also used in apexification procedures (5) and to prohibit the resorption activity (6). It has been reported that remnant Ca(OH)2 prevents the penetration of sealers into dental tubules and increases apical leakage (7,8), leads to changes in physical properties and sealing ability of canal sealers (9,10) and alters the setting of zinc-oxide-eugenol based sealers (11). Hence, it is important to completely eliminate Ca(OH)2 before root canal filling (12). The complex anatomy of the root canal may not allow this elimination using our conventional materials (13).

Numerous studies have shown a big interest in removing Ca(OH)2 from the root canal. They examined the role of different irrigation solutions (14) and devices for Ca(OH)2 removal from root canal using laser activated irrigation (15,16), sonic and ultrasonic activation of the irrigation (17,18), endodontic brushes (19), shaping rotary instruments (20), the RinsEndo system (Dürr Dental, Bietigheim, Germany) (21) and the EndoVac system (Discus Dental, Culver City, CA) with Self Adjusting Files SAF (Re-Dent-Nova, Ra’nana, Israel) (14). Many studies affirm the difficulty of removing all the Ca(OH)2 especially from the apical third of the root canal (19,21-23). A recent review of literature found that the ultrasonically activated irrigation is more efficient in removing Ca(OH)2 from the apical third than the apical negative pressure and the syringe irrigation (24).

**Figure 1:**
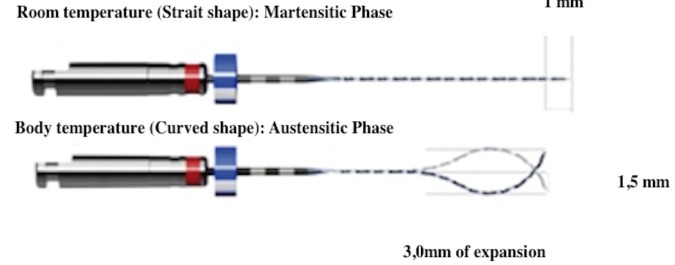
Description of the XP-Endo Finisher instrument.

The XP-Endo® Finisher (FKG Dentaire. La-Chaux-de-Fonds, Switzerland) is an instrument based on the shape-memory principles of the Ni-Ti alloy. This instrument is ISO 25 in diameter and zero taper. In its austenitic phase, the instrument has a curved tip and when rotating, it can achieve a diameter of 3mm in the last 10mm (Fig. 1). Therefore, the instrument has an improved physical contact with canal walls. The potential role of the XP-Endo® Finisher in removing the Ca(OH)2 has been assessed by a recent study (25) showing that there was no significant difference between the XP-Endo® Finisher and the passive ultrasonic irrigation PUI in removing the Ca(OH)2 paste from artificial grooves in the apical third of the root canal after one minute of activation. Although, one minute of working time is recommended by the manufacturer to optimize cleaning after shaping; removing Ca(OH)2 from the root canal may require more than one minute.

The aim of this study was to compare the efficacy of the XP-Endo® Finisher and the passive ultrasonic irrigation in removing Ca(OH)2 paste from the root canal, particularly from the apical third, after a prolonged period of activation. The null hypothesis tested in this study is that there is no difference between the two methods in eradication of the Ca(OH)2 paste from the root canals and from the apical third.

## Material and Methods

All phases of the study have received ethics approval from the Scientific Committee of the Faculty of Dentistry, Paul-Sabatier University, Toulouse-France. Sixty-eight extracted human mandibular incisors with complete apices, no radicular caries or visible cracks, were selected for this study. Radiographs of all the teeth were taken in a mesio-distal direction to confirm the presence of single canal. The teeth were decoronated to obtain the same length of all specimens 15mm. The glide path was established by introducing a K-file size 10 up to the apical constriction, the same instrument served to establish the apical patency when the tip of the file was seen out of the apex. The working length was determined to be 0.7 mm. short of the point where the K-file size 10 was first seen with a binocular Leica–WILD M3B (Leica Microsystems, Wetzlar, Germany) at X16 magnification. Two coats of different colored nail polish were applied to the teeth. The apical foramen was also sealed with the nail polish to prevent the leakage of the irrigant. Shaping was performed with BT-Race files (FKG Dentaire, La-Chaux-de-Fonds, Switzerland) with copious irrigation with 2.5% sodium hypochlorite and the canals were dried with sterilized paper points. A dressing of Ca(OH)2 (Produits Dentaires SA. Vevey, Switzerland) was prepared by mixing the pure Ca(OH)2 powder with distilled water, and was then placed in all the teeth using a paste-filler size ISO-25 except for four teeth, randomly chosen, which served as a negative control group. Radiographs of all the teeth were taken to control the quality of Ca(OH)2 filling. The coronal part of the canal was sealed with Cavit (3M ESPE, Seefeld, Germany). The samples were stored at 37° C and 100% humidity for 1 week.

Ca(OH)2 removal procedure.

Four teeth were chosen at random to serve as a positive control group; the Ca(OH)2 dressing was not removed from this group. The remaining sixty teeth were randomly divided, by using a 50-Cent coin, into two groups; G1: Xp-Endo® Finisher group (n=30) and G2: IrriSafe® group (n=30).

For both groups, after removing the coronal sealing, a size 15 K-file was introduced to the working length. The canal was then flushed with 2.5 mL of 2.5% sodium hypochlorite using a 27-gauge Monoject syringe. During this study, the injection speed was always 5 mL/minute and the needle (27-gauge Monoject syringe) was always inserted to one millimeter shorter than the working length. The master apical file BT3 was introduced, in respect of the working length of each sample, with a gentle watch-winding-pull motion and the canal was flushed again with 2.5 mL of 2.5% sodium. The canal was dried with sterilized paper points and was then rinsed with 1mL of EDTAT® (Acteon, Mérignac, France) which was left in the canal for three minutes. The canal was then rinsed with 2.5 mL of 2.5% sodium hypochlorite.

G1: XP-Endo® Finisher:

The XP-Endo® Finisher was used with an endodontic motor X-Smart (Dentsply Sirona, Ballaigues, Switzerland). The speed was set to 800 rpm and torque was set to 1 N.cm. Each tooth was held by a tight wooden pincer and placed in a controlled-temperature water path at 37°C without allowing water to enter the canal. The process of Ca(OH)2 removal consisted of three separate cycles. During each cycle, the XP-Endo® Finisher was carried to the working length with slow and continuous in-and-out movements of about 5mm amplitude for a period of one minute followed by the irrigation with 2.5 mL with 2.5% sodium hypochlorite. Finally, the canal was thoroughly dried with sterilized paper points.

G2: Passive ultrasonic irrigation PUI with IrriSafe® Size 20:

The IrriSafe® tips were used with ultrasonic unit P-Max Newtron® (Satelec®, Acteon, Mérignac, France) with the power set to 5. The IrriSafe® was inserted to one millimeter short of the working length and kept centered in the canal avoiding the contact with dentin walls. The procedure of Ca(OH)2 removal consisted of three separate cycles. During each cycle, the IrriSafe® tip was activated for a period of one minute followed by the irrigation with 2.5 mL with 2.5% sodium hypochlorite. Finally, the canal was thoroughly dried with sterilized paper points.

For both groups, the total operating time with each instrument was three minutes and the volume of the irrigation was 16 mL: 1mL of EDTAT® and 15mL of 2.5% sodium hypochlorite. Equal timings and amounts of irrigation were respected in this study.

Specimen preparation.

**Figure 2:**
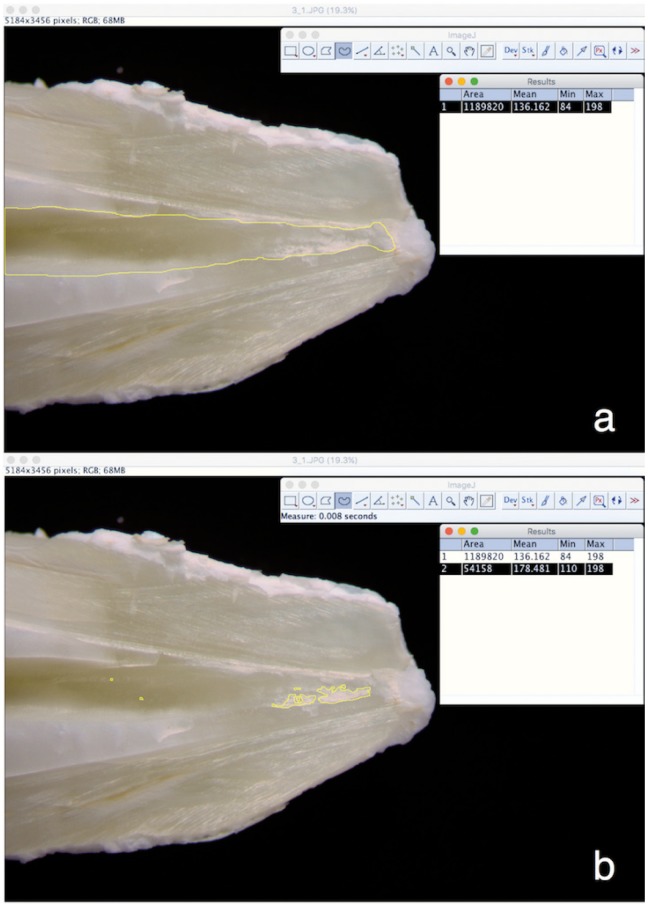
Calculation of the percentage of residual Ca(OH)2 using the ImageJ software. (a): Selecting canal borders. (b): Selecting areas covered in Ca(OH)2.

Grooves were prepared on the buccal and the lingual surfaces of each sample with a thin diamond bur used in water-cooled high speed hand-piece, keeping the dentin surrounding the root canal intact. The teeth were then split with a hammer and a small chisel. Photographs were taken of each half using a microscope (Leica-WILD M3B) at X6.4 magnification and a digital camera Canon® EOS 600D Digital SLR equipped with Tamron® macro (SP-90MM F/2.8 Di-VC-USD-1:1). The apical third was also captured at X16 and X40 magnifications using the same microscope and digital camera. The images were given random names to prevent the identification by the evaluators. The percentage of the remaining Ca(OH)2 in each half was calculated with the X6.4 photographs using the ImageJ program (The National Institutes of Health NIH, Bethesda, Maryland, USA). Two observers (The 1St and the 3rd authors) carried out several pixel-calculation tests with the ImageJ software to calibrate the calculation method. The calculation method was as following: Firstly, the canal borders were manually selected on the program which allowed the calculation of the root canal surface area in pixels. Then the total area covered in residual Ca(OH)2 was calculated by selecting the borders of all the individual patches of Ca(OH)2. In this way, it was possible to obtain the percentage of the canal surface covered by residual Ca(OH)2 was obtained (Fig. 2).

Photographs of the apical third at X16 magnifications were also coded to prevent the identification and were evaluated by two examiners (The 1st and the 3rd examiners) to give a score to each sample using the following scoring-system.

**Figure 3:**
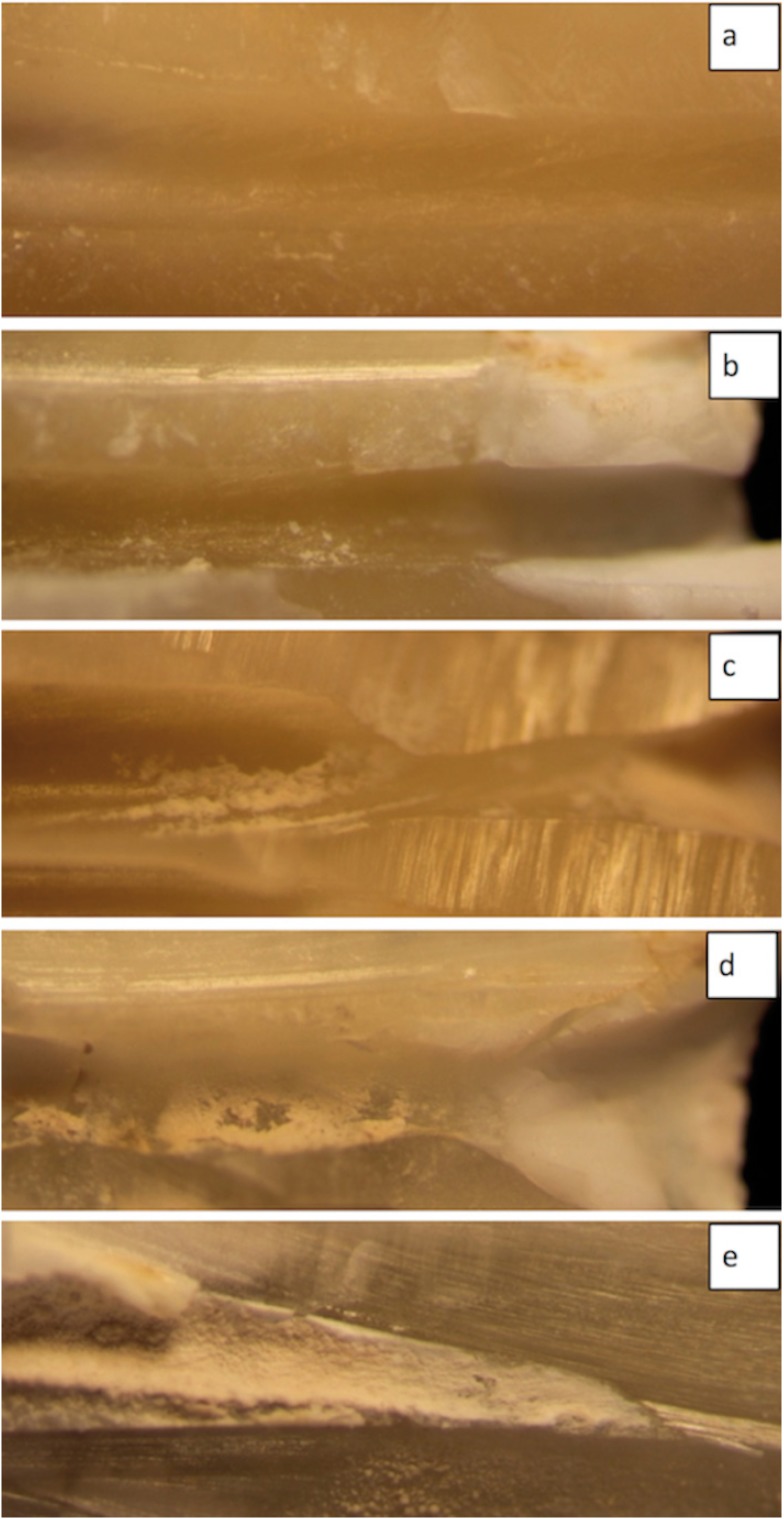
Apical third scores. (a) score 0: No Ca(OH)2 was found in the apical third. (b) score 1: Ca(OH)2 is covering less than 10% of the surface of the apical third. (c) score 2: Ca(OH)2 is covering less than 50% of the surface of apical third. (d) score 3: Ca(OH)2 is covering more than 50% of the surface of the apical third. (e) score 4: Ca(OH)2 is covering all the surface of the apical third.

0: No Ca(OH)2 was found in the apical third, 1: the Ca(OH)2 is covering less than 10% of the apical third, 2: the Ca(OH)2 is covering less than 50% of the surface of the apical third, 3: the Ca(OH)2 is covering more than 50% of the surface of the apical third, 4: the Ca(OH)2 is covering all the surface of the apical third (Fig. 3). Photographs at X40 magnifications were used to con-firm the scores of the apical third.

Statistical analysis:

The percentages of the surfaces with residual Ca(OH)2 were analyzed using the Wilcoxon-Mann Whitney test with *p* < 0.05. Kappa values were calculated to assess the inter-examiners agreement regarding the apical third scores. The efficacy of removing the calcium hydroxide dressing from the apical third was determined using the Fisher test with *p* < 0.05.

## Results

The XP-Endo® Finisher was able to completely remove the Ca(OH)2 from four teeth (13.33%) whereas the PUI was able to completely remove the dressing in one tooth (3.33%). The mean values for residual Ca(OH)2 were (2.1%) in the XP-Endo group and (3.6%) in the PUI group and this difference was not significant; *p*= 0.195. The Kappa values for the apical third scores were 0.79 and 0.76 for the XP-Endo® Finisher group and the PUI group respectively. The first examiner found the score 0 of the apical third in 14 samples of the XP-Endo® Finisher group whereas this score was achieved in 5 samples in the PUI group; *p* = 0.025. The second observer found the score 0 in 13 specimens in the XP-Endo® Finisher group and in 5 specimens in the PUI group; *p*= 0.047.

## Discussion

Previously published studies highlight the difficulty of the complete removal of the Ca(OH)2 pastes from the root canal system and from the apical third (14,22,24). The sonic activation of the irrigation results in significantly more remnants of Ca(OH)2 compared to the combination of rotary instrumentation and PUI (26). Numerous studies used the artificial groove model because it standardizes both the quantity of the Ca(OH)2 used and the dimensions of the groove (14,21). This model was not chosen in this study since it does not accurately represent the complexity of the root canal system (14). The quantity of the Ca(OH)2 placed in each canal was not standardized, canals were filled using a paste-filler and were then controlled radiographically. Ca(OH)2 removal was executed before separating samples. Hence, the efficacy of Ca(OH)2 elimination was assessed in more physiologically accurate conditions. Electron microscopic evaluation was not chosen as it is impossible to differentiate Ca(OH)2 residuals from the smear layer or dentinal debris (27).

The new recommendations for use of the IrriSafe® tips were adhered to this study; three one-minute cycles of activation were carried out for the PUI group and the same timings were also used for the XP-Endo® Finisher group. Moreover, the XP-Endo® Finisher was used in its austenitic phase at 37 °C in a temperature-controlled water path.

A recent study (25) has compared the PUI and the XP-Endo® Finisher for the eradication of the Ca(OH)2 from artificial grooves in the apical third; this study found that both methods were unable to completely remove Ca(OH)2 from the artificial grooves after one minute of activation and the difference was not significant. The current study proposes a prolonged activation period and an increased volume of irrigation for both methods of Ca(OH)2 elimination from root canals shaped by Ni-Ti instruments and not from artificial grooves; the XP-Endo® Finisher was able to completely remove the Ca(OH)2 from 4 samples (13.33%) whereas only one tooth (3.33%) was found to be Ca(OH)2 free in the PUI group. The mean values of remaining Ca(OH)2 were (2.1%) and (3.6%) in the XP-Endo® Finisher group and the PUI group respectively but the difference was not significant and the null hypothesis was accepted for the whole root canal but not for the apical third where the XP-Endo® Finisher showed superiority over the PUI and the difference was significant for both observers. The XP-Endo® Finisher has a small core “size 25” and zero taper. Due to its high flexibility, this instrument will contact and clean the dentine but not change the original shape of canal.

During this study, the XP-Endo® Finisher was carried to the working length in its austenitic phase with continuous slow in-and-out movements of around 5mm amplitude; the tip of the instrument was in contact with root canal walls in the last 5mm which correspond to the apical third. The design of this instrument allowed the complete cleaning of the apical third in 46.6% of the teeth for the first examiner, and in 43.3% for the second. The removal of the Ca(OH)2 is the consequence of the physical contact between the rotating instrument and the canal walls, especially in the apical third, but also consequence of the agitation of the irrigant due to its rotation at 800 rpm. Effective final irrigation protocol before root canal filling is indispensable to remove effi-ciently all the debris and the smear layer.

In the PUI group, our results match with previously published results concerning the difficulty in completely removing the Ca(OH)2 using the PUI tips (24,25). The acoustic energy delivered by the ultrasonic tip at frequencies of 25-30 kHz activates the irrigant solution and creates cavitation bubbles in the irrigant while the tip remains passive without contact with root canal walls. Therefore, the removal of the Ca(OH)2 dressing is the result of the cavitation phenomena which agitates the irrigation solution 

A new XP-Endo® Finisher-R, which has ISO-30 in diameter and zero taper, was recently introduced by FKG Dentaire, La-Chaux-de-Fonds, Switzerland. This instrument is designed to remove root filling materials adhering to the canal walls, especially in the curvature or oval areas. This new instrument may have an interesting role in removing the Ca(OH)2 dressing from the root canal due to its increased rigidity.

## Conclusions

Both methods investigated in this study were unable to completely remove the Ca(OH)2 from all specimens. The XP-Endo® Finisher was more efficient in removing the Ca(OH)2 dressing from the apical third than the PUI and this difference was significant.
